# Context-Tailored Food-Based Nutrition Education and Counseling for Pregnant Women to Improve Birth Outcomes: A Cluster-Randomized Controlled Trial in Rural Malawi

**DOI:** 10.1016/j.cdnut.2024.104506

**Published:** 2024-11-07

**Authors:** Penjani Rhoda Kamudoni, Lillian Kaunda, Marion Tharrey, Maggie Mphande, Shyreen Chithambo, Elaine Ferguson, Zumin Shi, Ibrahimu Mdala, Kenneth Maleta, Alister Munthali, Gerd Holmboe-Ottesen, Per Ole Iversen

**Affiliations:** 1 Department of Community Medicine and Global Health, University of Oslo, Oslo, Norway; 2 Department of Nutrition, University of Oslo, Oslo, Norway; 3 Department of Urban Development and Mobility, Luxembourg Institute of Socio-Economic Research, Esch/Alzette, Luxembourg; 4 Department of Precision Health, Luxembourg Institute of Health, Strassen, Luxembourg; 5 School of Nursing, Kamuzu University of Health Sciences, Lilongwe, Malawi; 6 Ministry of Health, Lilongwe, Malawi; 7 Department of Population Health, Faculty of Epidemiology and Population Health, London School of Hygiene and Tropical Medicine, London, United Kingdom; 8 Department of Human Nutrition, College of Health Sciences, QU Health, Qatar University, Doha, Qatar; 9 Department of General Practice, General Practice Research Unit, University of Oslo, Oslo, Norway; 10 School of Global and Public Health, Kamuzu University of Health Sciences, Blantyre, Malawi; 11 Center for Social Research, University of Malawi, Zomba, Malawi; 12 Department of Haematology, Oslo University Hospital, Oslo, Norway; 13 Division of Human Nutrition, Stellenbosch University, Tygerberg, South Africa

**Keywords:** dietary intakes, infants, low birth weight, Malawi, mothers, nutrition education counseling

## Abstract

**Background:**

Inadequate maternal dietary intakes remain a public health challenge in low-income countries like Malawi and can cause adverse birth outcomes.

**Objectives:**

To improve maternal dietary intakes and thus reduce the prevalence of adverse birth outcomes in rural Malawi.

**Methods:**

We performed a 2-armed (1:1) cluster-randomized controlled trial in Southern Malawi, enrolling pregnant women at gestational age 12–18 wk. Twenty villages (clusters) were randomly assigned to an intervention or a control group. A nutrition education and counseling (NEC) intervention consisted of education sessions followed by cooking demonstrations and counseling sessions. The women were encouraged to use locally available nutrient-dense foods to enhance dietary adequacy and -diversity. We applied linear programming to identify food combinations that could increase micronutrient intakes. The control group received standard antenatal health education.

**Results:**

Among the 311 women recruited, 187 (60%) completed the trial. We found no significant difference in mean birth weights recorded within 1 or 24 h of birth between the intervention and control groups. Intervention infants had greater birth length (*P* = 0.043) and abdominal circumference (*P* = 0.007) compared to controls, whereas other birth outcomes did not differ significantly. Notably, a quantile analysis revealed that the NEC intervention favored birth weight among mothers with a height below the mean height of the participant sample (156 cm) (*P*-interaction = 0.043).

**Conclusions:**

Tailoring NEC in food-insecure communities did not result in a significant difference in birth weight among infants of the participating mothers, but mean birth length and abdominal circumference were greater in the intervention group compared to controls. We noted that the NEC intervention favored birth weight among mothers with a lower height than the mean sample height. Our results warrant further investigation into offering tailored NEC early in pregnancy and on a larger scale.

This trial was registered at clinicaltrials.gov as NCT03136393.

## Introduction

Maternal and child malnutrition is a global public health challenge contributing to early child mortality, increased risk of disease, and compromised quality of life in adulthood [1,2]. Unprecedented efforts have rallied toward redressing child malnutrition and specifically averting stunting (low height/length-for-age and a common proxy for chronic undernutrition) [[3], [4], [5]]. However, efforts in addressing maternal malnutrition still lag behind, especially in low- and middle-income countries (LMICs) such as Malawi [6,7]. In line with this, our previous findings from observational and clinical studies showed that pregnant Malawian women had an inadequate dietary intake of various nutrients, and these data were also supported by biomarker analyses, e.g., of iron [8,9]. Importantly, adequate maternal nutrition is critical for optimal fetal development and presents the first window of opportunity for efforts to secure optimal growth [[10], [11], [12]].

Maternal nutrition during pregnancy is a key factor linked to infant birth weight [13]. It is a critical issue, especially in LMICs, where a high prevalence of micronutrient deficiencies among women of childbearing age is a public health challenge [14]. In line with this, infants born with a weight <2500 g [low birth weight (LBW)] face an increased risk of compromised growth and cognitive development as well as noncommunicable diseases later in life. According to the most recent national health survey [from 2016, i.e., when our cluster-randomized controlled trial (cRCT) was initiated], the prevalence of LBW in Malawi was 12%[15], whereas the most recent global and African prevalence of LBW was 14.7% and 13.9%, respectively, in 2020 [16]. Antenatal clinic (ANC) is the main source of nutrition education and counseling (NEC) for most Malawian pregnant women, and it is the most decentralized and accessible platform for the delivery of regular medical and nursing care. However, NEC offered through routine ANC rarely yields the anticipated nutrition behavior change due to low coverage. Furthermore, high client/provider ratios in the ANC clinics limit access to interactive or individual nutrition education sessions, and most women miss out as attendance at ANC remains low. These gaps necessitate the need for supplementary and focused NEC offered at the community level. NEC represents an intervention approach that, if shown to effectively improve dietary practices, can be scaled up in low-resource settings [17]. However, few studies have rigorously investigated its effectiveness in securing optimal maternal nutrition, and even fewer studies have investigated the effectiveness of theory-guided or context-tailored NEC in improving birth outcomes [18]. Moreover, successful implementation of NEC requires a structured approach (e.g., with the UNICEF framework for malnutrition) and often involves improved dietary behaviors, which may be facilitated by using frameworks (e.g., a socio-ecological framework and the theory for planned behavior) [19]. Theory-guided interventions showed a reduction in anemia when health education was provided to pregnant women in rural, nonagrarian, and food-insecure areas in Pakistan [20] and an increase in maternal weight gain among Indian pregnant women with a BMI (in kg/m2) lower than 18.5 [21]. Distribution of certain single or multiple micronutrient supplements during pregnancy reduces the incidence of LBW in LMICs [[22], [23], [24]]. However, the long-term sustainability of supplementation-based approaches in low-resource settings is often challenged due to both resource constraints and logistical limitations, including seasonal variations in agricultural outputs [25]. Complementary low-resource approaches, which can secure optimal maternal nutrition, are therefore needed.

We thus undertook a pragmatic cRCT where we examined the effect of a contextual tailored NEC intervention for pregnant women residing in rural Malawi on birth outcomes (birth weight, birth length, head circumference, abdominal circumference, and length of gestation). The NEC content was informed through the development of food-based recommendations (FBRs) to improve the nutrient adequacy of maternal diets in the study area. The intervention counseling and education approach was carried out by applying constructs of commonly used behavioral change theories. We hypothesized that tailored NEC would improve the pregnant women’s dietary intakes and, thus, the birth outcomes of their offspring.

## Methods

### Study site

The cRCT was undertaken in a sub-district (Nankumba Traditional Authority Area) of the Mangochi district in Malawi. Nankumba covers ∼150 km^2^ and has 150,000 inhabitants who reside in villages located on the western part of the shores of Lake Malawi. The inhabitants mostly live from subsistence farming and fishing.

### Approvals

This study was conducted according to the Declaration of Helsinki, and all procedures involving study participants were approved by the research ethics committee in Malawi (reference number P06/13/1406) and by the regional committee for medical and health research ethics in Norway (reference number 27884). Consent to participate was through signature or fingerprint. The study was registered with clinicaltrials.gov ID: NCT03136393.

### Study design

We randomly assigned clusters (i.e., villages) by applying an approach that would minimize “contamination” of the intervention activities between the clusters, given that the intervention was community-based and to be delivered through group activities. A cluster was thus designated as a village that did not share a border with any other village partaking in the study and was buffered from other villages surrounding any point of its border. In total, 20 out of 120 villages were selected and could qualify as clusters. We recruited all consenting primi- and multiparous pregnant women between their ninth and 16th gestational week who were available during the study period and planned to give birth at the health facilities within the study area. We excluded women carrying multiple fetuses and those with severe illnesses.

### Randomization and masking

The clusters were stratified into 2 strata based on their location (direct lakeshore and upper land). In each stratum, the clusters were randomly assigned into either intervention (*n* = 10) or control (*n* = 10) groups. The randomization (using random numbers generated from STATA software) was done by 1 of the researchers who did not have any role in the field implementation of the study. Data collectors and entry assistants were blinded vis a vis the 2 study groups to which a cluster belonged. We report the data according to the CONSORT guidelines.

### Study participants and power calculation of the primary outcome – birth weight

Eligible women living in the clusters were identified by community counselors and referred to the nearest local health facility where pregnancy and gestational age were confirmed, and then they were subsequently invited to consent to participate in the study. The study participants were pregnant women between 9 and 16 weeks of gestation (confirmed through ultrasound scanning at the local health facility). All consenting pregnant women, both primi- and multiparous, were eligible for inclusion. They had to be available during the study period and intend to give birth at the health facilities within the study area. Women carrying multiple fetuses and those with severe illnesses were excluded from the study. Recruitment lasted from 1 November, 2015 to 31 December, 2017. From a total sample size of 218, we estimated that a difference in mean birth weight of 150 g between the 2 groups could be detected, with a power of 80% and an alpha of 0.05 [26]. This was based on an intracluster correlation coefficient of 0.00001 calculated from findings from our pretrial studies on birth weight in the study area, where birth weight was measured utilizing the same methodology as in the current cRCT [27].

### NEC intervention development and implementation

The intervention was planned and developed prior to the cRCT through 6 steps.

In the first step, the dietary intake of pregnant women in the area was assessed to identify their risk of inadequate nutrient intake and generate the parameters used in the optimization modeling in the software “Optifood” [28]. This assessment was carried out prior to the cRCT. It showed that a high percentage of the women were at risk of inadequate intakes of zinc, iron, vitamin A, folate, vitamin B_6_, vitamin B_12_, niacin, riboflavin, and calcium [8].

In the second step, alternative sets of FBRs were selected using optimization modeling and the dietary parameters generated from the dietary assessment of pregnant women in the local area. These analyses showed that wholegrain maize flour, small-bony fishes, beans, orange sweet potatoes, mangoes, papayas, and milk were more frequently consumed nutrient-dense (mFCND) foods that could be promoted to improve dietary adequacy. If these recommendations were successfully followed, then most women following them would meet their nutrient recommendations. Furthermore, the optimization modeling indicated the minimum number of additional servings of each mFCND food that each pregnant woman would need to eat in addition to her typical meal.

In the third step, we mapped the most commonly used local recipes for the mFCND foods through key informant interviews and group cooking sessions in villages that were socioeconomically and culturally comparable to the study villages. Using participatory sensory evaluations (an iteration process), we modified commonly used meal and snack recipes such that a typical serving of the modified recipe could accommodate the earlier determined additional quantities of mFCND foods servings required to improve the nutrient adequacy of the diets of pregnant women in the area.

Thus, through steps 1–3, we crafted FBRs geared toward reducing the nutrient intake gaps in the area. As shown in Supplemental Table 1, these FBRs were promoted along with the general national dietary food guidelines.

The remaining steps of the intervention development were geared toward developing a counseling and education approach for the FBRs, which was informed by principles of commonly used behavior change theories that were sensitive to the sociocultural context of the area. Therefore, as a fourth step, we did a systematic literature review to identify commonly used frameworks and behavior change theories. These were the UNICEF framework for malnutrition, the theory of planned behavior, and the social-ecological model [19]. They were thus used to inform the counseling and education approach of the intervention. The selected theories provided guidance that the intervention should target not only the individual women but also significant others and key community persons (such as mothers-in-law, husbands, and health workers). In addition, based on the theories, we used goal setting in the counseling approach (e.g., adopting 1 FBR at a time within a week) and advised the women during the change process (e.g., to learn new food preparations).

As a fifth step, we explored the food culture, taboos, and norms in the area through an ethnographic formative study by a Malawian anthropologist and nutritionist (co-authors AM and PRK) to identify other modifications we would need in the counseling and education approach. Our approach was based on the following findings: day-to-day decisions in the households on what to eat were gender negotiated, with women being commonly responsible for farmed foods (staples and vegetables), whereas men oversaw the purchase of high-price fetching foods (animal source foods). The animal source foods would only occasionally supplement the staples and vegetables. This confirmed our earlier decision to include significant others, and we placed more emphasis on men than elderly women like mothers-in-law. Shared dish eating among all family members was the norm, which directed us to emphasize adding relishes as a separate dish for pregnant women.

In the sixth and final step, we trained lay health workers (LHW) in a series of 4 2*-*d trainings over 3 mo. During each training, 1–2 FBRs were focused on, the first day being a classroom training and the second day a cooking demonstration and role-play of adopting the FBR. It was mandatory that LHWs would practice the FBRs they had been trained on at home for their family meals before coming for the subsequent training. During the subsequent training, we discussed their own challenges in adopting the FBRs with the LHW and what could be useful in overcoming the challenges. The LHW’s own experience proved to be instrumental in assisting them better counsel the women when the intervention started. We held refresher training with the LHW, during which we continued discussing the obstacles encountered by the women in adopting the FBRs as well as problems encountered by the LHW in the counseling sessions.

The LHW worked with the study team to deliver the intervention through fortnightly (and later monthly) group (6–20 women) cooking demonstrations in each village. The intervention was delivered for a maximum period of 12 wk. The cooking demonstrations taught the women how to prepare foods that integrated the national dietary guidelines. Cooking demonstrations were done once a month, spending a whole afternoon and utilizing recipes that included ingredients made from food items turned into powders. The powders were used as seasoning to make the meals more diversified. The recipes combined foods with high content of nutrients that were lacking in their usual diet. The powders were from anchovy (Bonya, dried small bony fish), groundnuts (Nsinjiro), and dried moringa tree leaves (Chamwamba). Anchovies are a good source of calcium, iron, and zinc, groundnuts are a good source of proteins and fats, whereas moringa leaves are a good source of protein, vitamin B_2_, vitamin B_6_, vitamin C, iron, vitamin A, and magnesium. The diets in the communities participating in the study were predominantly plant-based and thus contained a high content of phytate and polyphenols that inhibit the absorption of iron, zinc, and calcium. The women thus got demonstrations on food processing that enables increased absorption of these nutrients, such as soaking beans overnight before cooking and soaking whole grain maize for 3 d before turning it into maize meals used for making porridge and the staple food Nsima. Prior to the cooking demonstrations, the women were assigned to prepare the powder ingredients, process maize flour, and mobilize all other resources needed.

We also offered home-based nutrition counseling to the women once a week to enhance intake according to the proposed dietary guidelines. The initial counseling sessions 1–4 focused on creating self-confidence in their capability to make the prescribed changes in diet and food preparation practices. The subsequent counseling sessions, 5 and 6, aimed at supporting the participating women in making decisions on setting goals for changing their eating patterns. The final counseling sessions focused on solving problems the participants would have been encountering in executing their goals. These counseling sessions were offered based on individual needs and involved women with their families, aiming to promote their capabilities and motivation to source the required ingredients. Each counselor was expected to support ≤20 women within walking distance of her house.

All study participants were encouraged to continue attending their scheduled sessions at their ANC during their pregnancy.

### Placebo education and counseling for the control group

To deal with a possible bias (in the expected effect of the NEC) due to the positive effect of being followed up, we offered nondietary counseling to the control group. The counseling was on health in general and repeated the nondietary aspects of ANC counseling. We did this also to mitigate the ethical dilemmas in selective intervention delivery inherent in the trial. The counseling was delivered in the same manner and number as in the intervention group for both group and individual sessions. In these sessions, goals were set by the participants on acquiring items they would need during childbirth (e.g., extra soap and basins) and saving money for skilled birth attendant delivery.

### Birth outcome measurements

Birth weight was measured by trained study nurses within 1 h of birth and repeated after 24 h. Birth weight (nude) was measured to the nearest gram using Seca 376 digital pediatric scales. Birth length was measured to the nearest 0.1 cm using a Seca 233 infantometer. We also measured the length of gestation (weeks) and placental weight (nearest gram). Measurements were assessed following the international fetal and newborn growth standards for the 21st Century and WHO standard procedures[29,30]. Given that women in the study area face challenges in timely access to health care at delivery, we provided all study participants with money for local public transport to their nearest health facility during the time of birth.

A precoded questionnaire developed and used in rural Malawi was administered at the first visit to collect sociodemographic variables at both participant and household levels, such as age, education level, occupation, number of children, number of people in the household, and food security. As a proxy for household economic status, a household asset index was calculated based on 11 household items given scores according to their monetary value [31].

### Statistical methods

We used descriptive statistics in the form of means with SDs and frequencies with percentages to summarize continuous and categorical variables, respectively. Mean differences between the groups were assessed using linear mixed-effects models adjusted for data clustering and gender of the child, whereas cluster-adjusted χ^2^ tests were used in assessing associations between categorical variables. We estimated the adjusted prevalence of LBW, preterm birth, and small-for-gestational age in both the control and intervention groups, with differences between the study arms assessed using adjusted risk differences. Simultaneous-quantile regression was used to examine the mean differences in various birth outcomes (birth weight, birth length, head circumference, abdominal circumference, and length of gestation) between the 2 study groups adjusted for gender with quantiles of interest set at 25, 50, 70, and 90. The Stata command used to conduct the simultaneous quantile regression was sqreg. We did not adjust for the cluster as the sqreg command in Stata does not have the cluster option.

## Results

**FIGURE 1:**
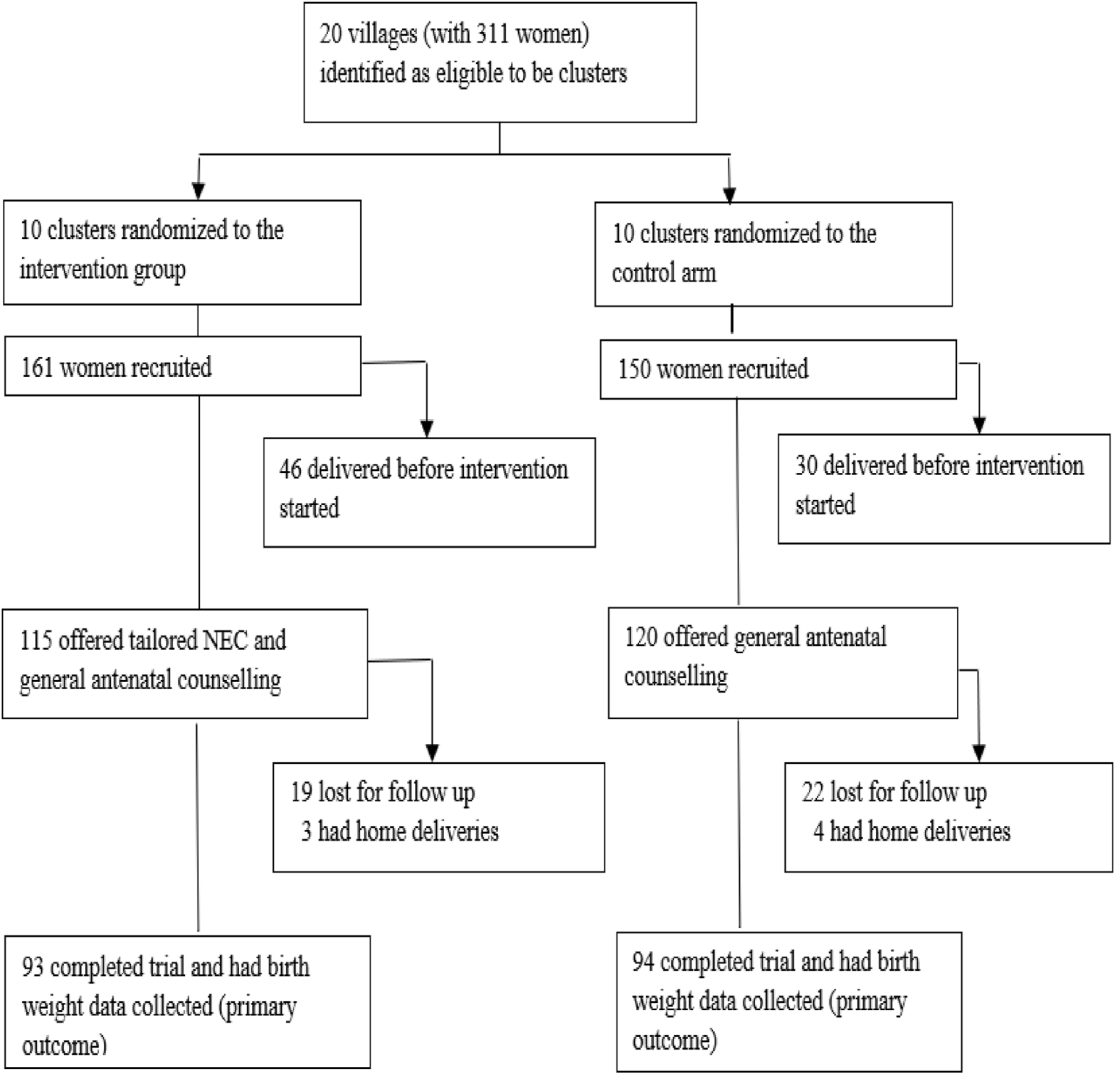
Flow chart showing the enrollment of the study participants into the randomized trial. NEC, nutrition education counseling.

The flow of study participants through the recruitment and randomization process is shown in Figure 1. We originally selected 20 clusters with a total of 311 women. In the end, 187 (60%) women completed the trial with the primary outcome (birth weight) recorded.

### Characteristics of the study participants

The anthropometric and sociodemographic characteristics of the study participants at baseline are shown in Table 1. The study participants who were lost before the trial end-point were not different in these characteristics compared with those who completed the study. The characteristics of women in the intervention group and those in the control group were also similar (Table 1), except that the latter group, on average, weighed ∼4 kg more.

**TABLE 1:** Baseline anthropometric and sociodemographic characteristics of the study participants.

	Number of women completing or not completing the study (lost)			Number of women by study group		
	Completed	Lost	*P* value^1^	Intervention	Control	*P* value^1^
	*n* = 187	*n* = 124		*n* = 94	*n* = 93	
Various [mean (SD)]						
Age (years)	24.7 (6.3)	24.6 (5.9)	0.979	24.9 (6.0)	24.5 (6.6)	0.656
Number of previous births	2 (1.9)	2 (1.8)	0.601	2.1 (1.7)	1.9 (2.1)	0.387
Weight (kg)^2^	55.0 (13.4)	55.8 (8.2)	0.567	53.0 (9.6)	57.0 (16.3)	0.041
Height (cm)	156 (10.4)	156 (15.6)	0.998	156 (13.4)	156 (6.2)	0.595
BMI (kg/m^2^)	22.5 (2.7)	26.2 (5.5)	0.360	21.8 (3.7)	30.1 (7.8)	0.001
Education [*n* (%)]			0.766			0.205
Illiterate	69 (36.9)	51 (41.0)		34 (36.2)	35 (37.6)	
Primary school	86 (46.0)	54 843.4)		48 (51.0)	38 (40.9)	
Secondary school	32 817.1)	19 (15.6)		12 (12.8)	20 (21.5)	
Household occupation [*n* (%)]			0.637			0.138
Subsistence farming	127 (68.0)	91 (73.1)		71 (75.5)	56 (60.2)	
Small-scale business	53 (28.3)	27 (22.0)		22 (23.4)	31 (33.3)	
Medium-scale business	7 (3.7)	6 (4.9)		1 (1.1)	6 (6.5)	
Type of housing			0.041			0.824
Grass thatched roof	170 (90.9)	102 (82.1)		85 (90.4)	85 (91.4)	
Iron-roof	17 (9.1)	22 (17.9)		9 (9.6)	8 (8.6)	
Domestic assets [*n* (%)]^3^			0.616			0.198
None	7 (3.7)	7 (5.7)		3 (3.2)	4 (4.3)	
Few assets	84 (45.0)	52 (41.5)		41 (43.6)	43 (46.2)	
Some assets	89 (47.6)	57 (46.39		49 (52.1)	40 (43.0)	
Enough assets	7 (3.7)	8 (6.5)		1 (1.1)	6 (6.5)	
Experienced food shortage last year [*n* (%)]			0.189			0.275
No	48 (25.5)	20 (15.6)		19 (20.4)	29 (30.8)	
Yes	138 (74.5)	104 (84.4)		75 (79.6)	64 (69.2)	

Abbreviations: BMI, body mass index; SD, standard deviation.

1 Cluster-adjusted χ^2^*P* values.

2 Weight was measured when included in the study, i.e., at various gestational ages.

3 Domestic assets included, e.g., television, radio, fridge, bicycle, mattress, mosquito net, fishing boat.

### Effect of the intervention on infant birth outcomes

No harm of the intervention was detected. Among the intervention women, 57% gave birth to a boy, and compared to the control women, 59% gave birth to a boy (*P* = 0.240). Neither the mean birth weight (primary outcome) recorded within 1 h of birth (*P* = 0.517) nor the mean birth weight recorded between 1 and 24 h after birth (*P* = 0.419) differed between the intervention and control groups (Table 2). The infants in the intervention group had a significantly greater birth length and a higher abdominal circumference compared to the controls, whereas birth weight and head circumference did not differ significantly between the 2 study groups. Notably, there was a nonsignificant trend toward higher values in weight and head circumference in the intervention group. In addition, there were no significant differences in placental weight or the length of gestation (Table 2).

**TABLE 2:** Effect of the intervention on mean birth outcomes and duration of gestation.

	Intervention (*n* = 73–94)^1^	Control (*n* = 66–93)^1^	Adjusted mean difference (95% CI)^2^	*P* value
Mean (SD) birth weight (g) within 1 h after birth	3016 (455)	2967 (480)	60 (–89 to 209)	0.408
Mean (SD) birth weight (g) within 24 h after birth	2913 (452)	2845 (394)	83 (–62 to 227)	0.243
Mean (SD) birth length (cm) within 1 h after birth	48 (3)	47 (3)	1 (0–1)	0.043
Mean (SD) head circumference (cm) within 1 h after birth	33 (14)	33 (13)	0 (–4 to 4)	0.976
Mean (SD) abdominal circumference (cm) within 1 h after birth	44 (20)	36 (19)	8 (–1 to 18)	0.007
Mean (SD) placenta weight (g) within 3 h after birth	584 (118)	568 (118)	7 (–42 to 56)	0.764
Mean (SD) length of gestation (weeks)	39 (3)	39 (3)	0 (–1 to 1)	0.696

Abbreviations: CI, confidence interval; SD, standard deviation.

1 The variable numbers are due to missing values.

2 Values are the least square mean differences between the intervention and control group and adjusted for baseline maternal weight, cluster, and sex.

In line with the conventional intervention impact analysis described above, when we performed a quantile analysis, the only birth outcome that differed significantly between the 2 study groups was abdominal circumference (at both the 50th and 70th quantile) (Table 3). However, there was a significant interaction between the mother’s height (below the mean of 156 cm) and intervention in relation to birth weight at 24 h (*P*-interaction 0.043) but not in relation to birth weight at 1 h (Supplemental Figure 1). A similar interaction was not noted for abdominal circumference, infant length (Supplemental Figure 2), or small-for-gestational length (Supplemental Figure 3).

**TABLE 3:** Effect of the intervention on birth size and duration of gestation using quantile analysis.

	Difference in quantiles between the intervention and control group			
	25th quantile	50th quantile	70th quantile	90th quantile
Birth weight (g) within 1 h after birth	125 (–68 to 318)	75 (–122 to 272)	127 (–16 to 270)	10 (–180 to 200)
Birth weight (g) within 24 h after birth	–1 (–283 to 281)	90 (–94 to 274)	19 (–160 to 198)	65 (–142 to 272)
Birth length (cm) within 1 h after birth	0.40 (–0.53 to 1.33)	0.10 (–0.63 to 0.83)	0 (–0.68 to 0.68)	–0.50 (–2.1 to 1.1)
Head circumference (cm) within 1 h after birth	1.0 (–6.9 to 8.9)	3 (–3.8 to 9.8)	2 (–4.0 to 8.0)	0 (–4.6 to 4.6)
Abdominal circumference (cm) within 1 h after birth	6 (–5.6 to 17.6)	9 (3.3–14.7)∗∗	11 (12–20.8)∗	8 (–1.9 to 17.9)
Length of gestation (weeks)	9 (–55 to 73)	12 (–31 to 55)	–13 (–57 to 31)	14 (–72 to 100)

Values are mean (95% confidence interval).

∗ *P* < 0.05.

∗∗ *P* < 0.01.

We next compared the proportions of LBW, preterm birth, and small-for-gestational age infants in the 2 groups. We could, however, not detect any statistical differences in these birth outcomes between the 2 study groups (Table 4). Interestingly, for both study groups, more infants were small-for-gestational age than LBW (Table 4).

**TABLE 4:** The effect of the intervention on low birth weight, prematurity, and small-for-gestational age.

	Intervention (*n* = 94)	Control (*n* = 93)	Adjusted mean difference^1^
Low birth weight^2^	10.6 (10)	14.0 (13)	33 (-6.1 to 12.8)
Preterm birth^3^	10.3 (10)	14.9 (14)	4.9 (-4.0 to 13.9)
Small-for-gestational age^4^	19.3 (17)	21.4 (19)	1.8 (-13.6 to 9.9)

Values are % (*n*).

1 Adjusted for sex of child.

2 Low birth weight is defined as birth weight <2.5 kg.

3 Preterm birth is defined as birth before gestational week 37.

4 Small-for-gestational age is defined as a birth weight <the 10th percentile for gestational age.

## Discussion

In the present study, we found that evidence-based tailoring of NEC to local dietary intake gaps in food-insecure communities did not result in a statistically significant increase in birth weight among infants of the participating mothers in the intervention group, nor did it change the prevalence of LBW infants, but the mean birth length and abdominal circumference were greater in the intervention group than in the controls. We also noted that the NEC intervention yielded a nonsignificant but consistent increasing trend in all parameters of birth outcomes. Unexpectedly, the intervention favored an increase in the mean birth weight of infants born to mothers with height below the mean height of the sample but not among infants born to taller mothers.

We have previously reported that the NEC had a positive effect on the dietary intake of women during pregnancy [32]. This finding is in line with the abovementioned nonsignificant trend of increased birth outcomes and may suggest that tailored NEC during pregnancy may have an effect on birth outcomes, warranting a randomized controlled trial (RCT) utilizing a larger sample as well as possibly a longer exposure to an NEC intervention.

The increments in birth weight reported from previous trials on interventions with lipid-based nutrient spreads in Malawi or with economic empowerment through conditional cash transfer are comparable to our observed increment in birth weight of ∼50 g [26,33]. This implies a probable comparability in the effectiveness of any of these interventions, all of which aim to complement the current ANC support. Moreover, the observed increase in birth weight reported here is the same magnitude that Ashorn et al. [26] reported in their RCT, where multiple nutrient supplementation or iron-folic acid supplements were given to Malawian women with uncomplicated pregnancies from <20 weeks of gestation. In our case, >3 quarters of the women received tailored NEC from the 14th week of gestation. Possibly, the observed effect could have been larger if the intervention had been offered earlier in pregnancy, as observed among women who had been exposed to the intervention longer; in these cases, the increment of birth weight was larger (data not shown). We experienced that it was feasible to recruit women in the first trimester by employing community counselors as a point of contact for recruitment as they were trustworthy and respected, and the women felt at ease to approach them. We would also argue that birth weight itself is not a decisive outcome in securing child health, but rather, the absence of morbidity and mortality is crucial. Nevertheless, birth weight can be a plausible indicator of both maternal nutrition and infant health.

The WHO, as well as the Ministry of Health Guidelines in Malawi for maternal nutrition during pregnancy, currently include the provision of iron-folic acid supplementation (through ANC). In our case, all women in the study (regardless of study group affiliation) were thus encouraged to continue attending ANC, where they were given these supplements. Furthermore, those in the control group received general community-based antenatal nutrition education. However, it is important to point out that the observed effect on birth weight probably pertains to the effect of NEC tailored to the local context and not generic nutrition education material. The tailoring of the NEC, in our case, focused on educating the women on a few modest but highly effective dietary changes that centered on increasing the consumption of locally available nutrient-dense foods in their diet. In contrast, nutrition education received in standard ANC typically educates women on all possible nutritious food combinations, including foods that are rarely eaten in their context. Thus, it is likely that nutrition information derived from ANC could be overwhelming for women who are negotiating food availability in their homes. In our case, the NEC information entailed achievable, practical changes that could be made using a few food items that were available in their setting.

The novel finding that shorter mothers (below the mean height of the participant study sample) apparently benefited more from the intervention in terms of birth weight is interesting. Generally, it is believed that short mothers are less likely to be able to provide adequate nutrition to the fetus during pregnancy, resulting in small infants who again may give birth to small infants in the next generation, as reviewed by Ozaltin et al. [34]. We speculate if the NEC intervention may have prevented this vicious cycle by improving maternal diets and thus positively impacting fetal growth. Possibly, then, one could identify a subgroup of such mothers and tailor-make a NEC specifically for them.

To our knowledge, at the time of undertaking the study, there were no RCTs that had investigated the effect of peer- counseling-delivered comprehensive nutrition education based on locally available foods, including cooking lessons on dietary diversity, birth weight, and maternal nutrition as an exclusive intervention in a low-income country context. In line with our findings on the effects of NEC on dietary intakes among our pregnant women [32], a recent Ethiopian RCT reported improved dietary diversity among pregnant women given nutrition education [35]. Similarly, public nutrition education tested in an RCT increased dietary intakes of micronutrients among pregnant Kenyan women [36]. Single studies have shown the effects of maternal education on birth weight. For example, a recent cRCT conducted in Indonesia found that a maternal mentoring program improved birth weight [37]. A recent cRCT from Ethiopia arrived at a similar conclusion [38]. In contrast, a recent systematic review and meta-analysis found no effect of nutrition counseling on birth weight in sub-Saharan Africa, but only 2 studies were included [39]. A large Bayesian network meta-analysis from 2019 of randomized clinical trials to improve birth weight in LMICs found no significant effect of maternal education; however, there were few RCTs included in the analyses, and they were heterogeneous in design and reporting of data [40].

The question of whether nutrition education, if made comprehensive and contextualized for local food environments, can improve dietary intakes among pregnant women as well as birth outcomes is crucial in the context of low-income countries such as Malawi. We would argue that the delivery of nutrition education, even when comprehensive, is less resource intensive and more sustainable relative to dietary supplemental interventions that are reliant on both financial resources for supplies as well as organizational resources for a functioning supply chain to safeguard against shortage of supplements. Thus, for struggling healthcare systems, comprehensive nutrition education is likely to be relatively easier to optimize and sustain over time once it has been set up. Notably, we previously reported improvement in behavioral mediators (reflecting more knowledge about nutrition) among women in the intervention group in our cRCT, and this was also translated into increased dietary diversity [32]. Another argument for comprehensive nutrition education offered to pregnant mothers is the expected translational improved effect on household nutrition as well. The skills that women are empowered with to improve their own nutrition can extend beyond giving birth, e.g., during breastfeeding and complementary feeding. Finally, the emerging epidemics of childhood obesity and other noncommunicable diseases in LMICs like Malawi necessitate a public health policy that supports healthy lifestyles, of which nutrition education may be a key factor.

The strength of our study is the pragmatic design combined with the methodological rigor of a cRCT, all set in a remote and low-resource study area. To develop the intervention, we were required to undertake a prestudy to estimate the percentage of women at risk of inadequate nutrient intakes and, therefore, nutrient deficiencies [8], as well as trying out the taste acceptability of dishes and conducting cooking classes for the women [28].

The weaknesses of the study were the loss of follow-up, yielding an underpowered sample, and a shorter-lasting intervention than planned due to the many challenges we had when conducting the RCT in a remote place like our study area with limited financial and logistical resources. We designed the study so that the duration of the intervention would last from enrollment (i.e., from gestational weeks 9–16) until birth with a full-term pregnancy of 40 wk, which should amount to a maximum of (40 - 9) = 31 wk and a minimum of (40 - 16) = 24 wk of intervention. As is evident from Figure 1, ∼40% of the women in the intervention group did not complete the whole study. Potentially, this could have affected the study results. Additionally, we usually were not aware of a woman’s pregnancy until the end of the first trimester or sometimes into the second trimester. Therefore, the period of exposure to the intervention was variable and, thus, sometimes shorter than expected. Moreover, we had no information about the mother’s prepregnancy weight.

In conclusion, the NEC examined in this study did not result in a significant increase in birth weight but greater abdominal circumference and length. However, the positive trend found in all birth outcomes warrants further investigation on a larger scale of the effectiveness of a tailored NEC offered early in pregnancy. If confirmed to be effective, in-depth regional studies of dietary patterns can be incorporated into the processes of developing or revising national dietary guidelines.

## Author contributions

The authors’ responsibilities were as follows– PRK: conceived and designed the study, performed the initial data analyses, and wrote the first manuscript draft; GH-O, POI: conceived and designed the study and interpreted the data; LK, MT, MM, SC: collected and interpreted the data; EF: in charge of optimization modeling in the software “Optifood” and interpreted the data; ZS, IM: responsible for the statistical analyses and interpreted the data; KM, AM: responsible for study design and interpreted the data; and all authors: read and approved the final manuscript.

## Funding

This project was partly funded by the Research Council of Norway, the University of Oslo, and Direktør Throne Holsts Fond for Ernæringsforskning, Norway. The funders had no role in the design, implementation, analysis, or interpretation of the data.

## Data availability

Data described in the manuscript will be made available upon reasonable request from the corresponding author.

## Conflict of interest

The authors report no conflicts of interest.
