# Supplementary material for: Context-Tailored Food-Based Nutrition Education and Counseling for Pregnant Women to Improve Birth Outcomes: A Cluster-Randomized Controlled Trial in Rural Malawi
Source: Curr Dev Nutr. 2024 Nov 7;8(12):104506. doi: 10.1016/j.cdnut.2024.104506 (PMC11626795; doi:10.1016/j.cdnut.2024.104506)

Supplementary Information:

Context-Tailored Food-Based Nutrition Education and Counselling for Pregnant Women to Improve Birth Outcomes: A Cluster-Randomized Controlled Trial in Rural Malawi

Penjani Rhoda Kamudoni et al.

Contents:

Supplementary Table 1-- Page 2

Supplementary Figure 1--- Page 3

Supplementary Figure 2--- Page 4

Supplementary Figure 3--- Page 5

Supplementary Table 1: Food-based recommendations of the nutrition education and counselling (NEC)

1- **National dietary guidelines' recommendations for dietary intakes during pregnancy**:

   a. Pregnant women should eat six food groups daily (staples, animal foods, fats, vegetables, fruits, legumes, and nuts)

2- **Food-based recommendations based informed by the intervention development process**:

   a. Increase intake of whole maize, dark green leafy vegetables (*molinga oleifera*), small bony fishes, groundnuts, beans, orange-flesh sweet potato to 5 – 7 times per week
      i. Double portion sizes of these foods except cereals or tubers
      ii. Adopt food preparation methods for these foods which could increase nutrient bioavailability i.e.
         a) Grains - Pre-germinate maize before home processing into flour
         b) Beans - Inclusion of an animal source food in cooking beans and intake with vitamin C-rich foods
         c) Dark green leafy vegetables - inclusion of oil during preparation
         d) Small bony fishes – Grounding them into powder to optimize intake of the bones as a source of calcium
      iii. Eat daily at least once, a multi-mix snack which could constitute whole grains and legumes and other foods (choose from recipes developed for the NEC)
      iv. Eat at least 3 meals (refer meal suggestion for the NEC) and two snacks a day

   b. Increase intake of milk (goat milk) to 2 – 3 times per week

Supplementary Figure 1: Effect of the intervention on infant birth weight in relation to maternal height, recorded 1 hour (A) or 24 hours (B) after birth.

A

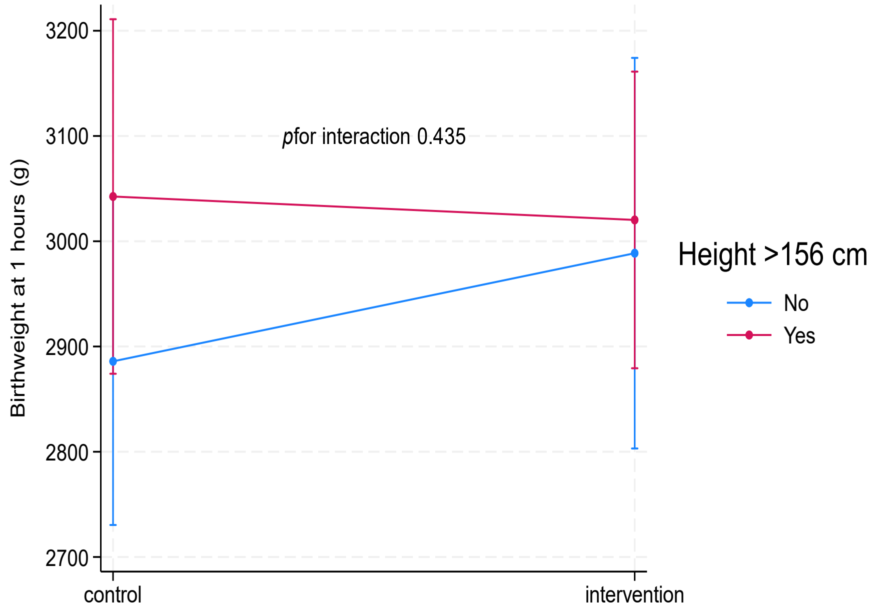

B

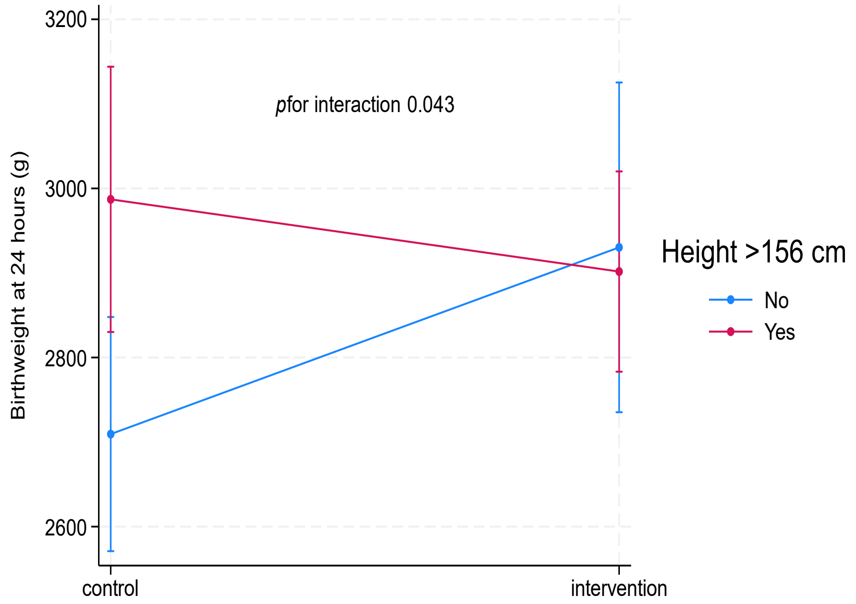

Supplementary Figure 2: Effect of the intervention on infant abdominal circumference (A) or infant length (B), in relation to maternal height.

A

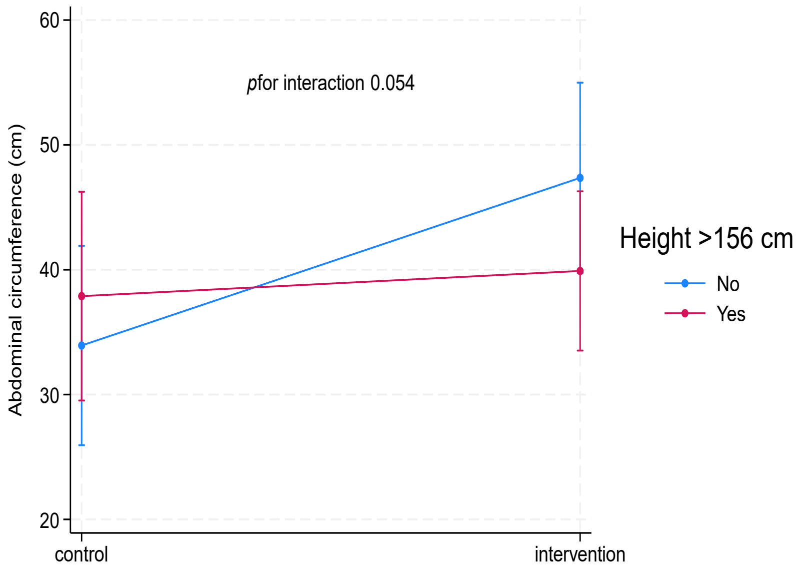

B

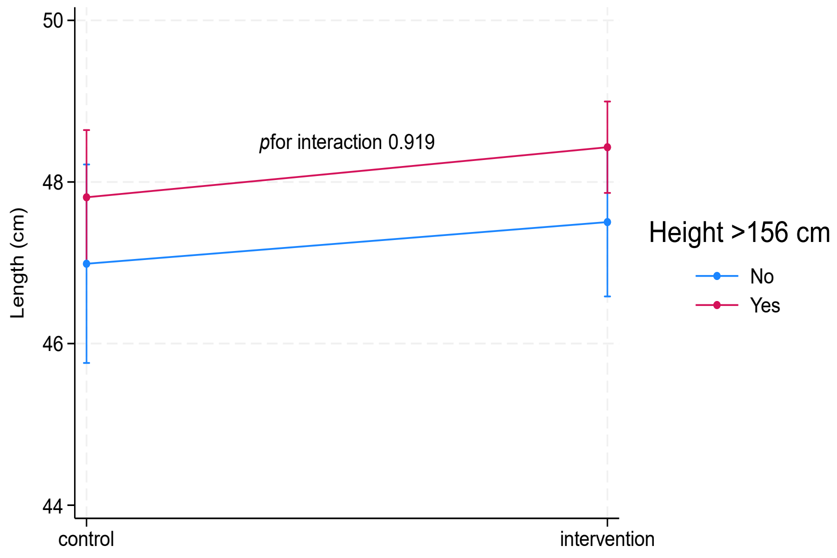

Supplementary Figure 3: Effect of the intervention on small-for-gestational age (A) or low birth weight (B), in relation to maternal height.

A

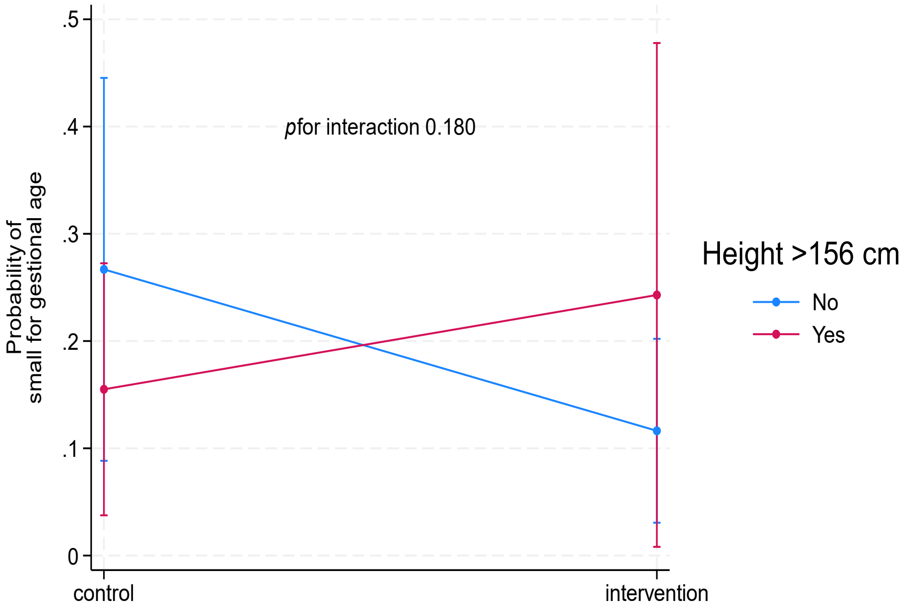

B

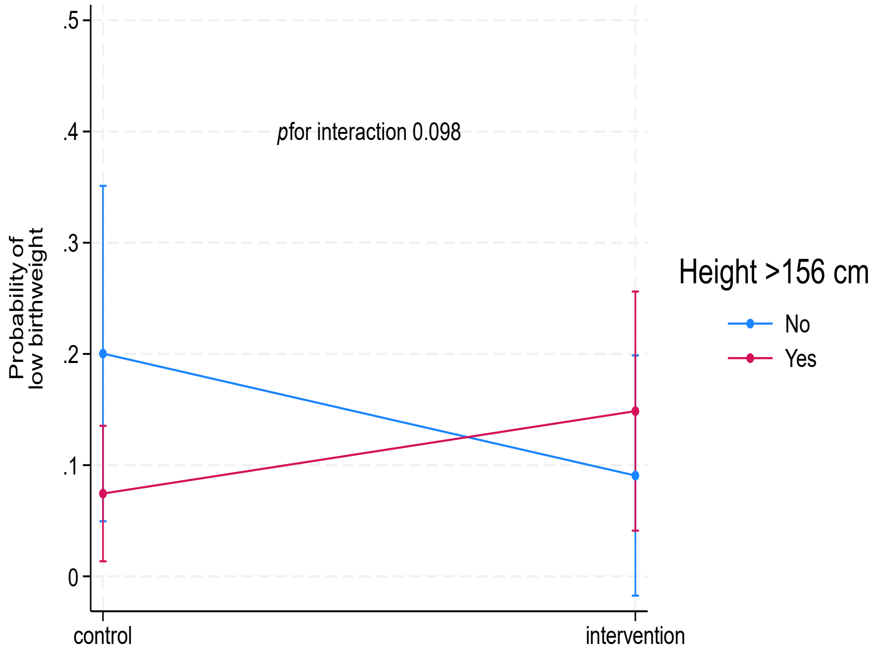

Supplement: Multimedia component 1 [file mmc1.pdf]
